# Implementation priorities in Australian community pharmacy: A semi-structured survey of Australian pharmacists

**DOI:** 10.1016/j.rcsop.2025.100683

**Published:** 2025-11-15

**Authors:** Veronika Seda, Stephen R. Carter, Rebekah J. Moles, Carl R. Schneider

**Affiliations:** The University of Sydney, Faculty of Medicine and Health, School of Pharmacy, Sydney, NSW, Australia

**Keywords:** Professional services, Implementation, Variation, Community pharmacy, Survey, Factors

## Abstract

**Background:**

Australian community pharmacy is undergoing transformation, with pharmacists increasingly contributing to primary care and providing professional services. Implementation science offers structured approaches to support the scalable and sustainable delivery of healthcare. However, most Australian research to date has been exploratory in nature.

**Objective:**

To identify key factors influencing the implementation of professional services in Australian community pharmacies and develop a community pharmacist implementation importance scale (CPIIS).

**Methods:**

A semi-structured national survey was conducted among Australian community pharmacists, guided by the Consolidated Framework for Implementation Research (CFIR) and Cochrane's twelve key implementation dimensions. Descriptive statistics summarised demographics and workplace practices. Exploratory factor analysis (EFA) examined perceived importance of implementation elements, and correlation analyses explored associations with pharmacist and practice characteristics.

**Results:**

Data from 108 eligible respondents were analysed. Pharmacists reported delivering over 9000 services in the previous 14 days, with dose administration aids (67 %) and absence from work certificates (55 %) being most common. EFA identified two key dimensions: “Inner” and “Outer” contexts, with internal factors, such as staffing, workflow, and resource availability, rated as more important. Pharmacist experience was associated with a decrease in the perceived importance of the outer context. The model demonstrated good fit and internal consistency.

**Conclusions:**

Australian community pharmacists currently provide an extensive number and range of professional services. Internal operational factors are prioritised for service implementation. CPIIS offers a practical tool to evaluate the priorities of community pharmacists for implementation of professional services.

**Ethics registration:** Ethics approval from the University of Sydney Ethics and Human Research Committee was obtained [2021/170].

## Highlights

• Community pharmacy services are expanding but implementation is variable.
• A new community pharmacist implementation importance scale was developed using implementation science frameworks.
• Two key dimensions emerged: “Inner” and “Outer” settings.
• Pharmacists prioritise internal factors like staffing and workflow over external ones.

## 1 Introduction

### 1.1 Professional services evolution

Community pharmacies have evolved from dispensaries providing products and basic advice, to healthcare hubs, offering a wide range of professional services. These professional services are often referred to as enhanced or extended,^1^ and are categorised into five domains: services for improving the quality use of medicines, product-focused services, primary care and public health services, harm reduction services and other advanced services, such as home care services, repeat dispensing or adjusting prescribed treatments.^2^ While the range offered had been relatively consistent for a long period of time in Australia, the COVID-19 pandemic further contributed to the expanded pharmacists' scope of practice through the introduction of new services.^3^ Therefore, the focus on quality, remuneration, and implementation has never been more important, so that patient communities can be provided with sustained support.

### 1.2 Professional services quality and remuneration

Across Australia, service-related quality assessment programs are optional and vary across pharmacy ownerships (see Box 1). Developments regarding quality assessment of service delivery have occurred in recent years. The most common program, utilised by pharmacy owners, was introduced in 1997 by the Pharmacy Guild of Australia (pharmacy owners lobby group). The Quality Care Pharmacy Program (QCPP), re-named Quality Care Program (QCP) in 2020, provides an independent, 2-yearly assessment of services offered. Pharmacy management and governance, consumer-centred care, human resources, premises, infrastructure and stock are cornerstones of the QCP assessment, in line with AS85000:2017 Quality Care Community Pharmacy Standard.^4^^,^^5^ As a result, pharmacy owners receive a certificate displaying the overall quality of the pharmacy business. Individual pharmacies can also develop their own internal quality assessments, but information is lacking.

Box 1 **Information about pharmacy ownership in Australia.6–8**

**Table:** 

Australian community pharmacy ownership facts
• Each Australian community pharmacy is owned by a pharmacist(s) as specified nationally by the Pharmacy Board of Australia.
• The pharmacy ownership in each state and territory is governed by its own legislation.
• Under the state and territory legislation, pharmacies may form a group under a unified trademark, brand or name, referred to as a ‘banner group’.

Alt-text: Box 1

Remuneration for any professional service delivered in community pharmacy is obtained from Australian federal or state/territory government, commissioned health providers, not-for-profit organisations, private insurance companies, or the patient/consumer.^9^, ^10^, 11., 12 Depending on the source of funding and location, service delivery varies across the country, highlighting the evolving scope of practice and variability between individual jurisdictions.13., 14, 15., 16, 17 Recently introduced services include administering any medication via injection beyond vaccines, for example, long acting buprenorphine, screening and treating urinary tract infections, and the provision of contraceptive pill to eligible persons. Scope of practice expansions are currently progressing in all Australian jurisdictions.18., 19., 20. This changing landscape calls for structured considerations regarding focus on the providers (pharmacists) and the system level (community pharmacies).^10^^,^^16^^,^^21^

### 1.3 Professional services implementation practices in Australia, focus on implementation science, context and implementation deliverers

Clinical outcomes of professional services in community pharmacies have been extensively studied in ‘controlled’ or ‘trial’ environments.^14^^,^^22^, 23., 24, 25, 26. However, the fact that clinical interventions have been successful in a trial or controlled environment, does not automatically guarantee sustainable and successful uptake in practice.^14^^,^27., 28., 29, 30, 31. The issue is historical, multifactorial and generally referred to as the ‘science to practice gap’.^30^^,^^32^^,^^33^ In the context of health services, implementation science is *“the scientific study of methods, which promote the systematic uptake of research findings and other evidence-based practices into routine practice, and, hence, to improve the quality and effectiveness of health services”* as stated by Eccles and Mittman.^34^ Implementation science evidence-informed methods encompassing theories, models and frameworks (TMFs) aim to ‘bridge’ this effectiveness to the implementation chasm.^35^^,^^36^ To accelerate the research translation process, implementation practices consider impact at multiple levels compared to traditional research, expanding its scope to the recipient (patient), provider (pharmacist), organisation (pharmacy or pharmacy organisation), and system (healthcare) levels.^33^ The long-standing implementation frameworks such as the Integrated-Promoting Action on Research Implementation in Health Services or iPARIHS or the Consolidated Framework for Implementation Research (CFIR) recognise the importance of these factors in sustaining long-term successful outcomes. They emphasise that people are central to facilitation efforts.^37^^,^^38^ The CFIR is considered as a determinant framework, which strength is in how it guides its users in systematically addressing implementation efforts, without missing contextual factors, including barriers and facilitators to implementation, which influence outcomes.^35^ CFIR is structured around five domains: Innovation Domain (Innov), Outer Setting Domain (OS), Inner Setting Domain (IS), Individuals Domain (Indiv), and Implementation Process Domain (IP). To expand on depth and specificity, each Domain is represented by multiple Constructs (*n* = 62) and Subconstructs (*n* = 19), see Table 1 for detail.^37^^,^^39^

**Table 1:** Consolidated framework for implementation research: Domains, constructs and brief descriptions (adapted from Damschroder et al).^37^

CFIR Domain	Number of constructs within a domain	Brief domain description
1. Innovation	Main constructs n = 8	The “thing” being implemented
2. Outer Setting	Main constructs *n* = 7 Subconstructs *n* = 3	The setting in which the Inner Setting exists
3. Inner Setting	Main constructs *n* = 11 10 subconstructs	The setting in which the innovation is implemented
4. Individuals	Roles subdomain: Main constructs *n* = 9 Characteristics subdomain: Main constructs *n* = 4	The roles and characteristics of individuals
5. Implementation Process	Main constructs n = 9 Subconstructs *n* = 6	The activities and strategies used to implement the innovation
CFIR Outcomes Addendum	Antecedent assessments: Main constructs *n* = 5 Implementation outcomes: Main constructs: n = 6 Innovation outcomes: Main constructs: n = 3	

There are many contemporary *qualitative* research articles focused on understanding perspectives of implementation of community pharmacist-delivered professional services in Australia.^1^^,^^26^^,^40., 41., 42, 43 However, the incidence of *quantitative* reports of the experiences of Australian pharmacists are at limited.^14^^,^^44^ For example, it is not known how many services are provided per unit of time, or the range of services provided, nor how much time is spent planning for service delivery. In addition, the extent to which pharmacists believe theory-derived elements of implementation are important for its success is unknown. Furthermore, there is a lack of understanding of the ways in which pharmacies utilise management practices to assist with implementation. Workplaces often use quality standards and procedure manuals and may establish formal processes for delegating or assigning responsibilities for service delivery. However, although these practices are generally assumed to occur, there is no published research on how widely they are actually implemented.

Considering both service *provider* (pharmacist) and *system* (community pharmacy) level, this study aimed to explore, describe and identify associations among factors influencing the planning and implementation of professional pharmacy services. At the service provider level *(community pharmacists)*, the objectives were to:

1. describe the demographic characteristics, professional background and workplace experiences,
2. examine the perceived importance of key implementation elements in the successful implementation of pharmacy services, and
3. investigate associations between pharmacists' characteristics and their perceptions of these implementation elements.

At the *system level (community pharmacies),* the objectives were to:

4. characterize the community pharmacies involved in delivering professional services, including their operational practices, and
5. identify associations between pharmacy characteristics and workplace practices related to service implementation.

## 2 Methods

An anonymous online, semi-structured (multiple choice, Likert-type response scale, open text response), cross-sectional survey, capturing qualitative and quantitative data, was administered to Australian registered community pharmacists from March to October 2022. The survey was selected as a research method due to its consistent nature, the possibility of online delivery (during the COVID-19 pandemic), and to reach pharmacists across metropolitan, rural and remote areas. Ethics approval from the University of Sydney Ethics and Human Research Committee [2021/170] was obtained.

### 2.1 Survey development

The survey was developed in three stages: evidence gathering, item generation, and content validation.

### 2.2 Evidence gathering

The evidence gathering stage consisted of searching relevant literature to ensure the survey was grounded in theory and contextualised to the Australian pharmacy services setting. CFIR was selected as the theoretical framework, because it is one of the most used implementation determinant frameworks in health services implementation, developed via a rigorous process, encapsulates over 100 original frameworks and developed through consultation with over 500 researchers and practitioners.^33^^,^^37^^,^^45^

In the context of this study, professional pharmacy services were defined as *services provided to a patient/carer inside a pharmacy by a registered pharmacist, beyond traditional dispensing and medicine counselling*. Therefore, to contextualise the Australian setting, reference was made to annual Australian Health Practitioner Regulation Agency (AHPRA) demographic survey results, Pharmacy Board of Australia's Policy requirements for practicing pharmacists, Quality Care Community Pharmacy Standards and the Seventh Community Pharmacy Agreement (see Table 2) to ensure the correct sample of participants were surveyed with recent and relevant experience in the implementation and/or delivery of community pharmacy services.

**Table 2:** Overview of survey questions' segments and evidence applied.

Survey segment	Question numbers	Total number of questions	Evidence applied in this segment (number of questions related)
Eligibility screening / Personal and pharmacy characteristics & Participants experiences with service delivery	1–12	*n* = 12	Annual AHPRA demographic survey (*n* = 8) Pharmacy Board of Australia's policy requirements for practising pharmacists (*n* = 1) Quality Care Community Pharmacy Standard (n = 1) Seventh Community Pharmacy Agreement (n = 1)
Service Implementation / participants' perceptions of importance of service delivery elements	13–70	*n* = 58	Cochrane's key dimensions of implementation (*n* = 25) Quality Care Community Pharmacy Standard (n = 2) CFIR (*n* = 19)
Demographics	71–75	n = 5	
Other	76	n = 1	Option to leave email for information about the study

### 2.3 Item generation

Survey items were generated for four categories of information: i) personal characteristics, used in the initial screening and also for demographic reporting, ii) participants' experiences of services delivery of a range of services, iii) participants' perceptions of importance of service delivery elements, and iv) pharmacy characteristics. Questions were initially developed by one research team member (VS) and subsequently reviewed by co-authors (CS, RM and SC). Discrepancies or disagreements were discussed, inconsistencies were resolved via consensus, and new terminology was applied. A detailed description of how the draft questions were changed or excluded is presented in Supplementary file 1. To elaborate, some participants' working background was elicited and retained from the screening questions (for those eligible). These included pharmacist registration (general, provisional, not registered), other qualifications and years of work experience.

After initial screening, participants were offered questions from the iv) pharmacy category. This was done deliberately to ground the participant to the setting in which services were provided. These questions were framed to describe/characterize the main pharmacy the participants worked in most, during the previous 14 days. These questions elicited: location of pharmacy (strip, shopping centre, stand-alone); level of rurality (metropolitan, regional, large rural town, medium rural town, remote community); membership of banner groups (versus independent); number of pharmacists and interns employed estimated as full time equivalents (FTEs); and average volume of prescriptions dispensed daily (n). There were questions aiming to explore the pharmacy's implementation processes, such as the use of various quality standards guides (y/n) (QCPP, staff-developed or banner group), the types of internal communication channels that were used (y/n) (verbal, meetings, notes in dispensing software, social media tools, and other forms), level of formality for how responsibility for services implementation was assigned (ranged from “0= no formal responsibility assigned”, “1 = assigned to the pharmacist on-duty’, 2 = “assigned to a particular pharmacist in the pharmacy”, 3 = “written into the employment contract”. In addition, a question asked (y/n) whether direct to patient charges for non-subsidised services were applied.

The next section of the survey included category ii) questions. These enquired about participant level of involvement in delivering specific professional services (absence from work certificates, blood pressure, Dose Administration Aids, Immunisation (COVID-19 and other), medication review services (MedsCheck, Diabetes MedsCheck), naloxone takeaway, opioid substitution, weight management, wound management and other services. These services were selected following a review of those listed in the current Australian Federal Government funding initiative – the Community Pharmacy Agreement).^46^^,^^47^ For each service, participants were asked to record within the last 14 days: the number of services delivered, number of service participants, an estimate of the maximum number of services delivered, and the amount of time spent planning for services (hours). A branching logic method was used for survey design such that participants responding to delivery of a particular service, were offered additional questions for that service. Therefore, participants who responded with more services were offered more questions.

In the next section, category (iii) questions were presented. There were 15 questions about participants' perceptions of the importance of service implementation elements for successful implementation of professional pharmacy services *in general*. The purpose of this section was to develop a scale for how participants rated the importance of service elements and to use this to draw associations between this scale and other factors derived from category i), ii) and iii) questions. The selection of elements was informed by Cochrane's twelve key dimensions of implementation and the CFIR domains and constructs.^37^^,^^48^ The implementation elements were initially based on the twelve key implementation dimensions developed by Cargo et al., with three additional items added following further consideration of CFIR (Supplementary File 1). These additions were specifically aligned with constructs related to successful service delivery planning, participant characteristics, consumer needs, organisational support, and outer settings influences.^37^^,^^39^^,^^48^ Items were adapted to suit the context of Australian community pharmacist-delivered professional services (performed by VS and CS). For example, it was considered that the terminology ‘dose delivered’ (of service) could be misinterpreted by practicing pharmacist respondents. For each element, an operational definition was written, and a corresponding questions (quantitative and qualitative) developed, asking participants to rate the importance of that element on a 5-point Likert scale (‘not at all important = 1’, ‘slightly important = 2’, ‘Important = 3’, ‘Fairly important = 4’ and ‘Very important = 5’, and to describe the particular dimension, see Supplementary file 2.^48^

The final section of the survey included category i) questions, regarding participants' demographic characteristics namely, gender, age and country of initial pharmacist registration. These questions were placed at the end following the expert panel's recommendation post content validity assessment.

### 2.4 Content validity

Before survey dissemination, content validity assessment (CVA) was performed with a group of Australian experts in pharmacy implementation science research and practice, who were recruited via purposive sampling.^49^^,^^50^ The inclusion criteria for the expert group were; experience in the fields of community pharmacy, and pharmacy practice and/or service implementation within Australia. A sample size of between eight to ten experts was chosen as per recommendations.^15^^,^^49^ Design and implementation of the CVA followed literature guidance^49^^,^^51^ and experts were asked to provide feedback on the service implementation segment of the survey.

Nine of ten invited experts ranked survey items using a three-point scale for essentiality (‘Essential’; ‘Useful, but not essential’; ‘Not essential’) during August to September 2021. The optional expert comments or suggested edits to the questions informed changes in the wording of the future survey questions for face validity. The overall content validity index (CVI) was 0.78.

### 2.5 Face validity

Following revisions which incorporated experts' recommendations to enhance items' clarity by one research team member (VS), these items were incorporated together with the screening eligibility and demographics. The updated survey was further face-validity tested by six pharmacists, whose feedback was implemented prior to survey deployment via the Research Electronic Data Capture (REDCap), which was used for survey dissemination and storage of participant's responses, see Supplementary file 3.^52^^,^^53^

### 2.6 Survey distribution

Convenience sampling and exponential non-discriminative snowball sampling methods were selected for this study phase.^54^ The intention of the survey distribution strategy was to gain views of pharmacists who represent all Australian jurisdictions and geographical areas with inclusion of both metropolitan, rural, and remote pharmacists, including pharmacists representing diverse age and practice experience. Eligible participants were community pharmacists currently practicing in Australian community pharmacy, holding a general or provisional registration with AHPRA. Pharmacists were recruited through email, social media posts, and at conferences. Invitations to participate were disseminated directly to these Australian community pharmacists via a link in an invitation email, social media post, or a flyer featuring a QR code, which led participants directly to the survey. Indirect dissemination was also utilised through professional pharmacy organisations' websites and newsletters. Non-practising pharmacists, students or pharmacists with limited AHPRA registration, and pharmacists having not practiced in community pharmacy for more than six hours a fortnight, were excluded from the study. The minimum sample size was calculated using publicly available data for the number of pharmacists practising in Australian community pharmacies (*n* = 19,200) at the time of calculation (1 March 2021). Using a precision sampling approach, we calculated that a sample size of 100 healthcare professionals was required to capture the perspectives of Australian community pharmacists. This calculation assumes 95 % confidence and a standard error of 5 %.^55^ While it was not technically possible to prevent multiple participation in this web-based survey, the likelihood of duplication appears minimal. To ensure anonymity while maintaining transparency, participants were given the option to request follow-up information about the study via a separate entry form. The majority of participants voluntarily provided their email addresses, and no duplicate email entries were observed. Based on this, the likelihood of multiple participation was considered minimal, and analyses were conducted under the assumption of unique responses.

### 2.7 Data analysis

The data obtained were extracted; descriptive and comparative statistics were conducted in MS Excel and SPSS. Missing data were excluded from the study and were not included in further analysis.^56^

### 2.8 Scale development with exploratory factor analysis

Since the dimensionality of a scale did not exist to measure how pharmacists perceive the importance of implementation elements, Exploratory Factor Analysis (EFA) was performed on the 15 perceived importance items using the guidance of El-Den et al.^49^ and reported according to Schreiber.^57^ Prior to conducting EFA, the suitability for factorability was determined using the Kaiser-Meyer-Olkin (KMO) where the aim was to exceed 0.8 or more.^58^ Given the sample size for this study was guided for representativeness, it was important to ensure that it was also sufficient for conducting EFA. While it has been shown that the suitability for EFA depends on a number of matters, it can be performed with samples as few as 50.^59^^,^^60^ However, most guidance generally requires 5 or more cases per item.^58^ Of critical importance was the presence of missing data generated because the response “can't answer” was recoded as missing.^56^ In cases where the data is Missing Completely At Random (MCAR), the most reliable results are obtained using imputation with Expectation Minimisation (EM).^59^ Little's test was non-significant, demonstrating that the data was MCAR. Data were then imputed using EM in SPSS v28, and EFA was performed using MPLUS v8.6 with a weighted least square mean and variance adjusted (WLSMV) estimator and oblique rotation. Inspection of the scree plot and parallel analysis was used to decide on the number of factors to retain.^61^ Items that had low loadings on the primary factor (<0.5) or high cross-loadings (>0.4) were excluded. Good model fit was determined by Root Mean Error of Approximation (RMSEA) less than or equal to 0.08 and Comparative Factor Index (CFI) higher than 0.95. Unweighted summative scales for perceived importance were created.^49^

Given the objectives of this study, this paper first presents results relating to *provider level* (pharmacists) in Part A and then in Part B the results relating to *system level* (community pharmacies). The report on survey findings in this paper aligns with the latest Checklist for Reporting Of Survey Studies (CROSS) recommendations.^62^ Applying this methodology to this article further enhances the quality of reporting on the survey overall, decreasing variability. The report on what recommended elements are included in this paper can be found in Supplementary file 4.

## 3 Results

### 3.1 Survey validation

Overall, 123 responses were obtained from respondents practising across Australian jurisdictions between March to October 2022. As there is no visibility how many pharmacists received the survey, the response rate is not estimable. After excluding responses with no data, students' survey responses and responses from pharmacists practising outside of community pharmacy, 108 (87.80 %) responses were analysed. The variation in the number of responses per survey section occurred. The length of the survey could have influenced the individual participants' varying levels of contribution with certain questions, where some respondents contributed selectively or omitted responses altogether, reflected in Table 3.

**Table 3:** Pharmacy characteristics and processes (*N* = 108).

Pharmacy characteristics			
	Category	n	%
Shopping precinct	Strip	47	43.5
	Shopping Centre	35	32.4
	Stand alone	23	21.3
	Not recorded	3	2.8
Level of rurality	Metropolitan	74	68.5
	Regional	7	6.5
	Large rural town	3	2.8
	Medium rural town	14	13
	Remote community	6	5.6
	Not recorded	4	3.7
Membership of banner group	Banner Group	75	69.4
	Independent	31	28.7
	Not recorded	2	1.9
Average number of pharmacists working (FTE)	1	21	19.4
	2	35	32.4
	3	28	25.9
	4 or more	23	21.3
Number of Intern pharmacists working (FTE)	0	52	48.1
	Less than1	3	2.8
	1	41	38
	2	8	7.4
	3	2	1.9
	4 or more	1	0.9
Volume of prescriptions dispensed	Median [IQR]	260 [160,400]	
	Not recorded	3	2.8

Pharmacy processes for professional services implementation			
Use of quality programs to guide service delivery	QCP (also known as QCPP)	73	67.6
	Staff developed programs	50	46.3
	Banner group developed programs	38	50.7
Level of formality for assignment of responsibility for services implementation	1 = No formal responsibility	9	8.3
	2 = Assigned to pharmacist on duty	30	27.8
	3 = Assigned to one pharmacist	29	26.9
	4 = Written into employment contract	7	6.5
	Not recorded	33	30.6
Internal communication channels for services information	Verbal communication	53	49.1
	Meetings	22	20.4
	Handwritten notes	63	58.3
	Notes in dispensing software	62	57.4
	Social media tools	48	44.4
	Other forms	10	9.3
	None recorded	25	23.1
	Number processes recorded Max, Median [IQR]	5, 3[1,4]	
Direct-to-patient charges	Paid	42	38.9
	Not paid	41	38
	Not recorded	25	23.1

### 3.2 Results Part A: A focus on providers – Objective 1

#### 3.2.1 Participants' characteristics

In the vast majority, 105 (97 %) of eligible survey respondents were community pharmacists holding general AHPRA registration, whilst 3 (3 %) were registered provisionally - intern pharmacists. Survey respondents practiced across all Australian jurisdictions, with the majority contributing to the survey from South Australia (37, 35 %) and New South Wales (33, 31 %).

Pharmacists advised of the following training completion: Immunisation training (36, 37 %), mental health training (24, 24 %), compounding (10, 10 %), medication management review accreditation course by the Australian Association of Consultant Pharmacy (AACP) (9, 9 %), among others. Training providers included professional pharmacy organisations (53 %), pharmacy or banner groups (35 %), or pharmacists who sought training themselves (12 %). The remaining demographic characteristics were only available from the 49 (45 %) participants that completed the whole survey. The majority of these participants identified as female (30, 61 %) and were aged between 27 and 76 years with a median [IQR] of 36^17^ years. The vast majority 46 (94 %) had obtained their initial qualifications in Australia. Participants had worked in pharmacy for a mean (SD) of 18 (14.67) years (see Table 3 for more detail).

#### 3.2.2 Participants' experiences with professional services delivery

In the last 14 days, 43 (40 %) of respondents reported they were involved in professional services for ten or more hours, while 36 (34 %) were engaged in professional services between one to five hours and 20 (19 %) between six to ten hours (Table 4). Eight (7 %) reported not being involved in professional services for a variety of reasons, such as services not being scheduled on the weekends, or other pharmacists' involvement in the services but not the respondents, no remuneration provided, hence no value seen and busy workload. Altogether, respondents reported the personal delivery of 9128 services in their past 14 days of practice. The most frequently reported services (by proportion of participants delivering them) were dose administration aids, which were delivered by 72 (67 %) participants, to a median [IQR] of 25 [4.57] patients. The second most frequently delivered service was issuing absence from work certificates by 59 (55 %) participants for a median of 2^1^^,^^5^ patients. The third was conducting blood pressure testing by 56 (52 %) participants for a median of 2^1^^,^^5^ patients. The least frequently delivered service was for Naloxone takeaway by 10 (9 %) participants for a median of 1 [0,3] patient. The delivery of a range of other services is reported in Table 4. The median [IQR] number of hours spent on planning each of the service types varied from 0.5 [0,1] for blood pressure management to 4^1^^,^^8^ for Dose Administration Aids (Table 4).

**Table 4:** Community pharmacist delivered professional services - Planning and delivery (*N* = 108).

Services provided	Participants involved in providing these services N(%)	Number of services to patients Median [IQR]	Hours Spent planning Median [IQR]
Dose Administration Aids	72 (67 %)	25 [4,57]	4 [1,8]
Absence from Work Certificates	59 (55 %)	2 [1,5]	1 [0,1]
Blood Pressure Management	56 (52 %)	2 [1,5]	0.5 [0,1]
Immunisations (Other than COVID-19)	55 (51 %)	10 [3,50]	2 [1,5]
MedsCheck (Medication use review)	48 (44 %)	5 [2,6]	1 [1,2]
COVID-19 Immunisations	46 (43 %)	15 [5,50]	1.5 [1,4]
Opioid Substitution	38 (35 %)	8 [2,20]	1[1,2]
Diabetes MedsCheck	36 (33 %)	2 [1,3]	1 [1,2]
Wound Management	31 (29 %)	2 [1,4]	0.5 [0.2,1]
Other Services	25 (23 %)	8 [3,20]	3 [1,9]
Weight Management	12 (11 %)	3 [1,5]	1 [0.25,3]
Naloxone Takeaway	10 (9 %)	1 [0,3]	0.75 [0,1]

#### 3.2.3 Participants' perceived importance of implementation elements – Objective 2

There was a high level of missingness in these items, with 33 participants not completing this section. This was attributed to the placement of these items towards the end of the questionnaire. These cases were removed from the EFA, leaving 75 cases with a low level of missingness (2.67 %). Table 5 reports participants' perceptions of the importance of each implementation element. The responses for all items were skewed towards the maximum level of importance, such that the median responses for most items were between 4 (important) and 5 (very important), except for Innov_02 which was 3.5.

**Table 5:** Perceived importance of implementation elements, relationship to CFIR domains and constructs.

CFIR		Code	Implementation element pharmacists rated in relation to importance (5-point Likert scale)	Median (IQR) responses
Domain (Subdomain)	Construct (Subconstruct)			
Innovation	Cost	Inn_01	The pharmacy's investment into the new service is financially viable	5 (1)
Innovation	Source	Inn_02	The new service you are about to start has been successfully trialed elsewhere	3.5 (1)
Outer Setting	Local Attitudes	OS_01	Patients return to the pharmacy because of this new professional service	5 (1)
Outer Setting	Financing	OS_02	The service is funded, e.g. someone pays for the service and is not provided by the pharmacy as ‘free of charge’	4.5 (2)
Outer Setting	Partnerships & Connections	OS_03	You are supported by external organisations in implementing this new service, for example by a professional organisation launching a promotional campaign	4 (2)
Outer Setting	Partnerships & Connections	OS_04	This new service considers partnerships with the local community	4 (2)
Outer Setting	External Pressure (Societal Pressure)	OS_05	The local community sees this new service as needed	4 (1)
Outer Setting	Partnerships & Connections	OS_06	Local health providers support the new service, e.g. if your pharmacy will be performing an eye screening, your neighboring optometrist and GP support this service	4 (2)
Inner Setting	Available Resources	IS_01	The amount of time the pharmacy staff has allocated to the service	5 (1)
Inner Setting	Culture (Deliverer-Centeredness)	IS_02	Your manager/s support you in implementing a new service	5 (0)
Inner Setting	Structural Characteristics (Information Technology Infrastructure)	IS_03	The service is supported by the IT technology in your pharmacy	5 (1)
Inner Setting	Structural Characteristics	IS_04	Before the new service is commenced, the pharmacy has the requisite equipment, such as necessary software, consultation room, operational procedures, materials, ready	5 (0)
Individuals (Characteristics)	Capability	Ind_01	The pharmacists and assistants have the requisite knowledge and clinical skills related to this service before it is implemented	5 (1)
Individuals (Characteristics)	Opportunity	Ind_02	The pharmacists practise to their full scope. NB: Expanded pharmacy services have been defined as working to their ‘full’ or ‘enhanced’ scope of practice, involving the performing of activities usually provided by other health professionals.	4 (1)
Implementation Process	Engaging (Innovation Recipients)	IP_01	The pharmacy has a plan, which outlines promotional activities for this new service	5 (1)

#### 3.2.4 Exploratory factor analysis

Prior to conducting EFA, the KMO statistic was 0.85 (meritorious) indicating suitability for EFA. Following imputation of missing data, EFA was conducted and there were 3 factors with eigenvalues above 3. However, the scree plot and parallel analysis supported a two-factor solution. The results of two-factor EFA indicated that three items OS_01, OS_02, IS_03 had low loadings (all below 0.4) and these items were subsequently excluded. The final solution is presented in Table 6. The Model fits the data adequately (RMSEA = 0.08 and CFI = 0.967) and there were no cross-loadings >0.4. Two factors were subsequently labelled “Inner context” and “Outer context”, “Inner context” included items probing participants' ratings of the importance of the pharmacy having the requisite investment in resources and manager support to enable service delivery. “Outer context” included items probing participants' ratings of the importance of the pharmacy having external support and engagement with local communities in order to enable service delivery. Overall, participants tended to rate elements in the “Inner context” with higher importance than “Outer context” CFIR domains.^37^ Unweighted summative scales for each factor were created with good internal consistency (Cronbach's alpha = 0.85 and 0.81, respectively). Overall, factor loadings were high (0.51–0.92). This finding, combined with the low number of factors, good number of items per factor, and good model fit, suggest that the derived factors have a low likelihood of biased factor loadings. The perceived importance of inner and outer setting factors were moderately correlated (*r* = 0.473, *p* < 0.01). Discriminant validity between the factors was established since the observed correlation was below 0.85.^63^

**Table 6:** Exploratory factor analysis of perceived importance of implementation elements (*N* = 75).

Factor	Code	Item	Factor loadings	
			1	2
Factor 1 Inner context	IS_04	Before the new service is commenced, the pharmacy has the requisite equipment	**0.918**	−0.061
	Inn_01	The pharmacy's investment into the new service is financially viable	**0.855**	−0.115
	IS_02	Your manager/s support you in implementing a new service	**0.731**	0.337
	IS_01	The amount of time the pharmacy staff has allocated to the service	**0.653**	0.170
	IP_01	The pharmacy has a plan which outlines promotional activities for this new service	**0.634**	0.042
	Ind_02	The pharmacists practice to their full scope.	**0.538**	0.400
	Ind_01	The pharmacists and assistants have the requisite knowledge and clinical skills related to this service before it is implemented	**0.536**	0.201
Factor 2 External context	OS_03	You are supported by external organisations in implementing this new service	−0.121	**0.915**
	OS_06	Local health providers support the new service	0.147	**0.748**
	OS_04	This new service considers partnerships with the local community	−0.002	**0.728**
	Inn_02	The new service you are about to start has been successfully trialed elsewhere	0.017	**0.549**
	OS_05	The local community sees this new service as needed	0.295	**0.513**

#### 3.2.5 Associations between pharmacists' characteristics, service experiences and perceived importance of implementation elements – Objective 3

A correlation matrix of associations between these variables is presented in Table 7. Participants that provided a wider range of services were more likely to also provide a higher number of services (0.369, *p* < 0.01). Participants' factor scores derived from the ‘outer’ perceived importance factor were negatively correlated with increasing experience (*r* = − 0.483, p < 0.01). There were no other significant associations.

**Table 7:** Relationships between pharmacists' characteristics, service experiences and perceived importance of implementation elements.^1^, ⁎

	Gender	Experience^2^	Rurality^3^	Range of extra qualifications^4^	Number of services provided 5	Range of services provided^6^	Time spent planning^7^	Importance (Internal context) factor score^8^
Experience^2^	0.011							
	47							
Rurality 3	0.140	0.131						
	44	46						
Range of extra qualifications^4^	0.028	0.102	−0.130					
	46	48	104					
Intensity of service delivery 5	−0.210	−0.250	0.030	−0.030				
	34	36	79	81				
Range of services provided^6^	−0.284	−0.111	−0.174	0.051	**0.369**^**⁎⁎**^			
	35	37	80	82	**80**			
Time spent planning^7^	−0.047	0.073	−0.117	0.022	0.013	0.016		
	36	38	80	82	60	61		
Importance (Internal context) factor score^8^	−0.115	−0.249	−0.040	0.018	0.054	−0.070	−0.110	
	30	32	72	74	56	57	55	
Importance (External context) factor score^8^	−0.268	**−0.483**^**⁎⁎**^	0.007	0.107	0.210	0.239	−0.039	**0.473**^**⁎⁎**^
	30	**32**	72	74	56	57	55	**74**

1 Spearman's correlation (two ordinal variables), point-biserial correlation (ordinal vs dichotomous) and phi (two dichotomous variables) (** P < 0.01) (N = number paired correlations).

2 Years of practice (n.b. age was also collected but was highly correlated with experience and was not reported here).

3 Measured using the Modified Monash model (Range, 1 = metropolitan to 6 = remote community).

4 Sum of the number of extra qualifications reported (Range, 0 to 5).

5 Number of services provided to individual patients in last 14 days (Range, 0 to 2634).

6 Range of different services provided in last 14 days (Range, 1–12).

7 Hours spent planning all services provided in last 14 days (Range, 0–220).

8 Unweighted Factor score (average score for each of the items in the factor) (Range, 1–5) Higher scores indicate higher level of perceived importance of internal/external factors in service delivery.

⁎ Response rates varied across different sections of the survey, resulting in variable sample sizes for each analysis. No imputation or interpretation of missing data was undertaken; analyses were conducted using available data for each output, and sample sizes are reported accordingly.

### 3.3 Results Part B: A focus on system level

#### 3.3.1 Community pharmacies' characteristics – Objective 4

Pharmacists reported practising in pharmacies that were in a variety of precincts, including being within a strip of shops, 47 (44 %), shopping centers, 35 (32 %), and a variety of other settings (Table 8). A majority, 75 (70 %) worked in banner group pharmacies and dispensed a median [IQR] of 260 [235] prescriptions per day. Just 21 (19 %) reported working in a pharmacy with 1 full-time equivalent pharmacists (FTE), 35 (33 %) employed with 2 FTE, while 51 (48 %) employed 3 or more FTEs. Pharmacy interns were employed in 52 (49 %) of the pharmacies.

**Table 8:** Relationships between pharmacy practices related to service delivery.^1^, ⁎

	Prescript-ion volume^2^	Average number of pharmacists working (FTE)^3^	Average number of Intern pharmacists working (FTE)^3^	Use of QCPP^4^	Use of staff-developed procedures^4^	Use of banner-developed procedures^4^	Level of formality for assignment of responsibility for services implementation^5^	Range of internal communication channels for services information^6^
Number of pharmacists working	**0.587**^**⁎⁎**^							
	**105**							
Number of Intern pharmacists working	**0.326**^**⁎⁎**^	**0.325**^**⁎⁎**^						
	**105**	**107**						
Use of QCPP	−0.088	−0.043	0.067					
	105	107	107					
Use of staff-developed procedures	0.062	0.127	0.148	**0.286**^**⁎⁎**^				
	105	107	107	**108**				
Use of banner-developed procedures	−0.027	0.083	0.049	**0.644**^**⁎⁎**^	**0.325**^**⁎⁎**^			
	74	76	76	77	**77**			
Level of formality for assignment of responsibility for services implementation	0.166	**0.255**⁎	0.076	−0.128	−0.109	−0.217		
	72	**74**	74	75	75	49		
Range of internal communication channels for services information	0.041	0.08	0.067	**0.597**^**⁎⁎**^	**0.536**^**⁎⁎**^	**0.423**^**⁎⁎**^	**−0.268**⁎	
	105	107	107	**108**	**108**	**77**	**75**	
Pharmacy charges private payments for services^7^	−0.07	0.1	0.031	0.078	0.182	0.114	−0.046	0.323**
	83	83	83	83	83	61	60	**83**

1 Spearman's correlation (two ordinal variables), point-biserial correlation (ordinal vs dichotomous) and phi (two dichotomous variables) (* *P* < 0.05, ** *P* < 0.01) (N = number paired correlations).

2 Average daily number of prescriptions dispensed.

3 Average number of pharmacists (and/or interns) employed in Full-time equivalents (FTE)s.

4 Use of Quality Care Pharmacy Program (QCPP), staff-developed or banner-developed procedures for service delivery (ye/no).

5 Range (0 = No formal responsibility, 1 = Assigned to pharmacist on duty, 2 = Assigned to one pharmacist, 3 = Written into employment contract).

6 Range (0 = no communication channels to 6 = all communication channels).

7 Yes/no.

⁎ Response rates varied across different sections of the survey, resulting in variable sample sizes for each analysis. No imputation or interpretation of missing data was undertaken; analyses were conducted using available data for each output, and sample sizes are reported accordingly.

#### 3.3.2 Pharmacy work practices relating to professional services

Regarding the use of quality programs to guide service delivery, 73 (68 %) used QCP, while 50 (46 %) used programs developed by staff and 38 (51 %) used programs provided by banner groups. Participants reported using a wide range of communication channels (Table 8). The median [IQR] was = 3.^1^^,^^4^ The most frequently reported channel was handwritten notes (63, 58 %), followed by notes in dispensing software (62, 57 %) and verbal communication (49 %). In regard to how the delegation of responsibility for services implementation was assigned, 9 (8.3 %) reported assigning no formal responsibility, 30 (28 %) assigned responsibility to a pharmacist on duty, 29 (27 %) assigned services to one pharmacist in the pharmacy, while 7 (6.5 %) assigned responsibility through a written employment contract.

#### 3.3.3 Relationships between pharmacy characteristics and work practices – Objective 5

A correlation matric of associations between these variables is presented in Table 8. Unsurprisingly, higher prescription volume was highly associated with high numbers of pharmacists (*r* = 0.587, *p* < 0.01) and interns (*r* = 0.325, p < 0.01) working at the pharmacy. A higher average number of staff working was associated with increasing formality of arrangements for assigning responsibility for service delivery (*r* = 0.255, *p* < 0.05), but no other pharmacy process. The use of QCP was associated with higher staff-developed (*r* = 0.286, *p* < 0.01), and (*r* = 0.644, p < 0.01) banner developed procedures for service delivery. The use of QCP (*r* = 0.597, p < 0.01), staff-developed (*r* = 0.536, p < 0.01) and banner-developed procedures (*r* = 0.423, *p* < 0.01) were associated with the use of a wider range of internal communication processes. Using a wider range of internal communication processes was also associated with the pharmacy being more likely to charge private fees for services (*r* = 0.323, p < 0.01) and negatively associated with increasing formality of arrangements for assigning responsibility for decisions around service delivery (*r* = − 0.268, *p* < 0.05). Somewhat predictably, delivering a wider range of services was correlated with the number of services delivered (*r* = 0.369, *p* < 0.01). There were no other significant associations.

## 4 Discussion

This study had employed a semi-structured survey as a method and EFA to investigate factors influencing planning and implementation of professional pharmacy services in Australia. This study addresses both service provider (pharmacist) and system (community pharmacy) levels, using an evidence-informed survey design to explore, describe and identify associations among implementation factors. Findings reveal that Australian community pharmacists, had a strong focus on internal implementation elements and limited attention to external influences. Study results highlighted opportunities for service implementation improvement through broader frameworks, clearer roles, and better communication. A community pharmacist implementation importance scale (CPISS) was developed to guide service planning, implementation, and delivery.

This study offers valuable insights into the implementation practices of Australian community pharmacists, marking a novel contribution through its use of a survey tailored to both provider and system levels and stages of implementation. The application of internationally recognised implementation frameworks, such as CFIR and Cochrane's twelve key dimensions of implementation, added theoretical depth and ensured comprehensive coverage of relevant domains. The survey instrument, which included the importance elements scale, was carefully adapted to the pharmacy context and validated for content and face validity, enhancing its relevance and clarity. Respondent demographics aligned with national data from AHPRA, lending credibility to the sample. Importantly, the study identified practical opportunities for improvement in service delivery, including the need for enhanced internal communication, broader training beyond clinical skills, and more structured implementation processes.

However, the study also presents limitations that should be acknowledged. The study was limited to Australian pharmacists, with a relatively low response rate and incomplete submissions due to survey length, potentially affecting the robustness of the findings. The reliance on self-reported data introduces bias and limits objectivity, while the exclusive focus on Australian pharmacists restricts generalisability to other contexts. Additionally, some key domains of the implementation framework, such as innovation characteristics, individual roles, and the implementation process were underrepresented in responses, suggesting a gap in awareness or emphasis among participants. The absence of methodological triangulation (in addition to the survey) further limits the depth of analysis, highlighting the need for future research using complementary approaches to validate and expand upon these findings.

The emphasis placed by pharmacist respondents on the importance of inner setting implementation factors aligns with existing literature exploring implementation of professional services in community pharmacy.^64^, ^65^, ^66^ For example, Mustafa et al. examined the implementation of the MedsCheck service in Malaysian community pharmacies based on CFIR and Australian service model. They found that pharmacists' strong focus on inner setting may have reduced pharmacists' communication skills and confidence. To address low public awareness, pharmacists relied on inner setting's service provision to help generate demand for MedsChecks.^64^ A recent systematic review by Nowak et al. used the CFIR framework to expore implementation of opioid substitution services in community pharmacy, emphasising the critical role of inner setting for successful uptake. They also acknowledged the importance of engaging community collaborators and reaching outer setting stakeholders.^65^

The reduced priority to domains such as innovation and individual characteristics through our survey findings contrasts with studies that highlight these elements as critical implementation drivers.^67^, ^68^, ^69^, ^70^ These differences may reflect the tailored terminology and structure of the survey, as well as the specific context of Australian community pharmacy practice.

This study suggests that Australian community pharmacists to prioritise internal, practice-level factors—such as staffing, workflow, and procedural standardization when implementing professional services, with decreased emphasis on external influences including community partnerships, funding mechanisms, or broader health system pressures. This internal focus may reflect the realities of busy pharmacy environments and the immediate operational challenges pharmacists face. For clinicians and policymakers, these findings highlight a potential opportunity to support pharmacists not only through clinical training but also by strengthening capabilities in service design, stakeholder engagement, and implementation science. Tailored support in these areas could help bridge the gap between policy intentions and on-the-ground service delivery.

However, these findings should be interpreted with caution. The study does **not** imply that pharmacists are unaware of or uninterested in external factors. Nor does it suggest that an internal focus is inherently problematic. Rather, it points to a pattern that may be shaped by context, experience, and existing support structures. The limited response rate and reliance on self-reported data mean the results may not be fully generalisable, and further research is needed to explore whether similar patterns exist across different settings and among pharmacists who are leading innovation in their communities. Policymakers should be careful not to overgeneralise these findings but instead consider them as a starting point for more targeted inquiry and support.

While this study sheds light on the priorities of Australian community pharmacists in service implementation, several questions remain. For instance, it is unclear why pharmacists rank innovation-related constructs—such as effectiveness, evidence base, and affordability—relatively low in importance. Further investigation is needed to understand whether this reflects a lack of exposure, perceived irrelevance, or systemic barriers to innovation adoption. Additionally, the limited emphasis on individual characteristics and roles raises questions about how personal attributes, leadership styles, or professional identity influence implementation success.

Future research could explore these gaps through qualitative methods with an implementation science focus, which may uncover deeper motivations, contextual nuances, and lived experiences not captured in survey data. Comparative studies across different healthcare settings or countries could also help determine whether the observed internal focus is unique to Australian community pharmacists or part of a broader phenomenon. Moreover, longitudinal research could assess how pharmacists' implementation perspectives evolve over time, especially in response to policy changes, training interventions, or shifts in healthcare delivery models. Finally, exploring the role of external stakeholders, such as consumers, funders, and local health networks, in shaping service sustainability, would provide a more holistic view of implementation dynamics.

## 5 Conclusions

This study has identified key factors associated with successful planning and delivery of professional services in Australian community pharmacies. The nationally recognised Quality Care Program (formerly QCPP) plays an influential role in shaping operational practices within Australian community pharmacies. The survey results reveal that pharmacies adhering to QCP/QCPP standards are more likely to implement additional protocols and standard operating procedures (SOPs). The QCP/QCPP serves as a foundational framework for broader quality and compliance initiatives.

Another key finding is that pharmacies with a more structured approach to assigning responsibilities also have streamlined communication channels. This reduction in communication complexity is interpreted as a sign of operational efficiency, potentially decreasing the need for intensive supervision and monitoring. Such efficiency is particularly relevant in contexts where pharmacies are transitioning towards service-based models, including billing for professional services, where clear and concise communication pathways are essential.

Together, these insights underscore the value of structured quality frameworks and organisational design in successful implementation of professional services, supporting both regulatory compliance and service innovation.

## Authors' contribution

VS contributed to study conceptualisation and methodology, project administration, writing – original draft, review and editing, writing, visualisation, investigation, delivery, analysis and interpretation of data, statistical analyses, drafting of the manuscript, and critical revision of the manuscript for important intellectual content. RM contributed to conceptualisation and methodology, delivery, analysing and interpreting data and critical revision of the manuscript for important intellectual content. SC contributed to the conceptualisation, analysis, interpretation of data and writing – review and editing of the manuscript for important intellectual content. CS contributed to the study conceptualisation and methodology, acquisition, analysis and interpretation of data, statistical analyses, writing – original draft, review and editing of the manuscript, and critical revision of the manuscript for important intellectual content. VS and CS had full access to all the data in the study and take full responsibility for the integrity of the data and for the accuracy of the data analysis. VS (corresponding author) attests that all listed authors meet the authorship criteria and that no others meeting the criteria have been omitted. All authors read and approved the final manuscript.

## Authors' information (optional)

VS is a part-time PhD candidate at The University of Sydney School of Pharmacy, Faculty of Medicine and Health, The University of Sydney and a Clinical Advisor, Implementation Science Unit at the Department for Health and Wellbeing, South Australia.

RM is a Professor at The University of Sydney School of Pharmacy, Faculty of Medicine and Health, The University of Sydney.

SC is a Associate Professor at The University of Sydney School of Pharmacy, Faculty of Medicine and Health, The University of Sydney.

CS is an Associate Professor at The University of Sydney School of Pharmacy, Faculty of Medicine and Health, The University of Sydney.

## CRediT authorship contribution statement

**Veronika Seda:** Writing – review & editing, Writing – original draft, Visualization, Project administration, Methodology, Investigation, Formal analysis, Data curation, Conceptualization. **Stephen R. Carter:** Writing – review & editing, Visualization, Validation, Supervision, Methodology, Investigation, Formal analysis, Data curation, Conceptualization. **Rebekah J. Moles:** Writing – review & editing, Methodology, Investigation, Formal analysis, Conceptualization. **Carl R. Schneider:** Writing – review & editing, Writing – original draft, Validation, Methodology, Investigation, Funding acquisition, Formal analysis, Conceptualization.

## Consent for publication

There is no individual person's data, hence no consent for publication has been sought from the participants.

## Ethics approval and consent to participate

The study obtained ethics approval from the University of Sydney Ethics and Human Research Committee [2021/170]. All participants, which data we include in this study gave their written consent to participate in the study.

## Declaration of generative AI and AI-assisted technologies in the writing process

During the preparation of this manuscript, the authors utilised Grammarly and Microsoft Copilot (enterprise edition) to enhance language clarity and readability. All content generated or refined using these tools was subsequently reviewed and edited by the authors, who take full responsibility for the final version of the manuscript.

## Funding

This research did not receive any specific grant from funding agencies in the public, commercial, or not-for-profit sectors.

## Declaration of competing interest

The authors declare that they have no competing interests or financial interests or personal relationships that could have appeared to influence the work reported in this paper.

## Data Availability

The data captured in this study and the way it is managed has been approved by the University of Sydney Ethics and Human Research Committee. The data is saved on University Sydney drives, protected by password. The data collected in this study is anonymous.

## Appendix A Supplementary data

Draft questions, excluding demographics and screening questions Image 1

Adaptation of the twelve key dimensions of implementation by Cargo et al to pharmacy context Image 2

Mapping the implementation of professional services in Australian community pharmacy questionnaire Image 3

Checklist for Reporting Of Survey Studies (CROSS) Image 4
