# Supplementary material for: Implementation priorities in Australian community pharmacy: A semi-structured survey of Australian pharmacists
Source: Explor Res Clin Soc Pharm. 2025 Nov 15;21:100683. doi: 10.1016/j.rcsop.2025.100683 (PMC12686930; doi:10.1016/j.rcsop.2025.100683)
Supplement: Supplementary file 1 — Draft questions, excluding demographics and screening questions [file mmc1.docx]

**Supplementary File 1.** Draft questions, excluding demographics and screening questions.

| Proposed Question to the expert panel | Draft question number for expert panel | I-CVI (clarity)) | Excluded? | Reason for exclusion/Framework applied/Comment/Rewording  If related, then to which question in the final survey |
| --- | --- | --- | --- | --- |
| How is the pharmacy different from other pharmacies in professional services delivery? | 1 | 0.11 | Yes | I-CVI |
| What professional pharmacist services were you involved in? | 2 | 0.33 |  | Unchanged  Q12 |
| For those services selected in Q2: How many patients did you deliver *this service* to? | 3 | 0.33 |  | Edited for planning only Q13, |
| For those services selected in Q2: On average, how much time did you spend on the service per patient (considering planning, delivery, documentation, referral, evaluation)? | 4 | 0.67 |  | Q57 |
| For those services selected in Q2:  How confident did you feel when providing *the service*? | 5 | 0.78 |  | Unchanged  Q14 |
| What is the process this pharmacy uses to communicate to staff about this professional service(s), in general, most? | 6 | 0.22 |  | Edited as per recommendations  Q15 |
| If it was up to you, which of the following services listed below is the service you would prefer to provide above all? | 7 | 0.56 |  | Edited as per recommendations  Q16 |
| Why have you prioritised this service? | 8 | 1.00 |  | Unchanged |
| Have patients ever paid directly for professional service(s) in the pharmacy you are thinking about? (i.e. the one you worked most in the last 14 days). | 9 | 0.56 |  | Edited as per recommendations  Q18 |
| Please explain the process the pharmacy used to charge for a service(s). | 10 | 0.44 |  | Edited as per recommendations  Q19, merged together with q12 |
| Why do you think the pharmacy does not charge for services? | 11 | 0.67 |  | Edited as per recommendations  Q20 |
| When thinking about the professional service you delivered most in the community pharmacy, what type of payment is more acceptable for this service? | 12 | 0.44 | Yes | Excluded and incorporated into  Q19, merged together with q10 |
| Please think about the service you provided most. Does the pharmacy have a standard operating procedure (SOP) for the service? | 13 | 0.67 |  | Edited as per feedback  Q49 and associated Q61 |
| Please comment further, if you wish | 14 | 1.00 |  |  |
| Before any of the services were provided, was there any piloting or testing performed? | 15 | 0.44 |  | Edited as per feedback  Q23 |
| How was this achieved? | 16 | 0.63 |  | Edited as per feedback  Q24 |
| Does the pharmacy have a 'risk management plan' for the service(s) they provide? | 17 | 0.56 | Yes |  |
| Please comment further, if you wish | 18 | 0.83 |  |  |
| How important is the *pharmacy's marketing strategy* to you? | 19 | 0.56 |  | Cargo et al - Edited as per feedback  Q60 |
| How important is the *financial return on investment* to you? | 20 | 0.67 |  | CFIR - Edited as per feedback  Q26 |
| How important is*increasing customer's loyalty* to you? | 21 | 0.78 |  | CFIR - Edited as per feedback  Q25 |
| How important is *whether the new service is evidence-based to you*? | 22 | 0.78 |  | CFIR - Edited as per feedback  Q28 |
| How important is *the amount of time pharmacy staff have allocated to the service* to you? | 23 | 0.44 |  | CFIR - Edited as per feedback  Q29 |
| How important is the *staff's skillset* to you? | 24 | 0.33 |  | CFIR - Edited as per feedback  Q30 – merged with Q30 into the new Q30 |
| How important are *community needs* to you? | 25 | 0.44 |  | CFIR - Edited as per feedback  Q31 |
| How important is it *that the pharmacy is pre-equipped for the service* to you? | 26 | 0.56 |  | CFIR - Edited as per feedback  Q32 |
| How important is it *that the new service is accepted by local health providers (GPs, optometrists, etc)* to you? GPs represent general practitioners | 27 | 0.67 |  | CFIR - Edited as per feedback  Q33 |
| How important is it *that the pharmacists practise to their full scope* to you? | 28 | 0.56 |  | CFIR - Edited as per feedback  Q34 |
| How important is *the support from your leadership, i.e. operations manager, owner* to you? (If you already are a leader, how important is the management's support to you?) | 29 | 0.44 |  | CFIR - Edited as per feedback  Q35 |
| How important is that *the training support is available* to you? | 30 | 0.56 |  | CFIR - Edited as per feedback  Q30 |
| How important is it that *the service provision* *integrates with current* *software* to you? | 31 | 0.67 |  | CFIR - Edited as per feedback  Q36 |
| How important is *external support/advice* to you? | 32 | 0.22 |  | CFIR - Edited as per feedback  Q37 |
| How important is that *the service has an impact on the local community* to you? | 33 | 0.56 |  | CFIR - Edited as per feedback  Q38 |
| How important is it *that the service is* reimbursed (paid for)? (e.g. by a patient, by another party) | 34 | 0.67 |  | CFIR - Edited as per feedback  Q39 |
| Who decides on the services to be implemented? | 35 | 0.89 |  | CFIR - Small edition, almost unchanged |
| Are any of the pharmacy staff members assigned to be 'in charge' of the service? | 36 | 0.67 |  | CFIR - Edited as per feedback  Q41 |
| Who is this person? | 37 | 0.78 |  | CFIR - This and 38 merged into the new Q42 |
| Is this person in charge of multiple professional services? | 38 | 0.89 |  |  |
| What are the main tasks of such a pharmacist who is assigned one or more professional services in the pharmacy? | 39 | 0.44 | Yes |  |
| The pharmacy is considering launching a new service. How is this service, in general, delivered? | 40 | 0.44 |  | CFIR - Cochrane - Edited as per feedback  Q43 |
| The pharmacy is considering launching a new service. How do they 'make room' for this initiative? | 41 | 0.38 |  | Cargo et al - Edited as per feedback  Q44 |
| 1. Maximum number of patients participating in the service per unit of time | 42 | 0.44 |  | Cargo et al - Edited as per feedback  Q45 |
| 2. How many patients received the service | 43 | 0.50 |  | Cargo et al - split  Q47-48 |
| 3. How many patients fit the criteria for the service | 44 | 0.67 |  | Cargo et al - Edited as per feedback  Q59 |
| 4. How many patients were approached per type of recruitment strategy, e.g. pharmacist approached, flyers handed | 45 | 0.25 | Yes |  |
| 5. Steps of the service delivery, e.g. standard operating procedure (SOP), and how the pharmacist follows from the procedure | 46 | 0.56 |  | Cargo et al - Edited as per feedback  Q49 |
| 6. Changes to the service by pharmacist who delivers the service, which increased quality and/or effectiveness of the service | 47 | 0.44 |  | Cargo et al - Edited as per feedback  Q62 |
| 7. How many patients received the service elsewhere | 48 | 0.38 |  | Cargo et al - Edited as per feedback  Q63 |
| 8. How many ineligible patients received the service or should have received the service, but did not | 49 | 0.44 |  | Cargo et al - Edited as per feedback  Q48 |
| 9. Patient satisfaction survey/interview | 50 | 0.78 |  | Cargo et al - Edited as per feedback  Q53, 65 |
| 10. Pharmacist satisfaction survey/interview about the service they delivered | 51 | 0.75 |  | Cargo et al - Edited as per feedback  Q54, 66 |
| 11. What support the pharmacist receives from close colleagues regarding the service delivery | 52 | 0.50 |  | Cargo et al - Edited as per feedback  Q55, Q67 |
| 12. What support the pharmacist receives from allied health care providers in relation to the service delivery | 53 | 0.50 |  | Cargo et al - Edited as per feedback  Q56, Q68 |
| You considered a maximum number of patients participating in the service per unit of time. How would you estimate this number? | 54 | 0.56 |  | Cargo et al - Edited as per feedback  Q57 |
| You considered how many patients received the service. How would you estimate the number? | 55 | 0.67 |  | Cargo et al - Edited as per feedback |
| You considered how many patients fit the criteria for the service. How would you estimate this number? | 56 | 0.78 |  | Cargo et al - Edited as per feedback |
| You considered how many patients were approached per recruitment style, e.g. pharmacist approached, flyers handed over, etc. How would you record the recruitment style? | 57 | 0.50 |  | Cargo et al - Edited as per feedback |
| You considered steps of the service delivery (protocol or plan, checklist) and how many steps the pharmacist followed from the protocol. How would you develop the protocol? Tell us about the steps in more detail, please. | 58 | 0.56 |  | Cargo et al - Edited as per feedback |
| You considered changes to the service by a pharmacist who delivered the service, which increased the service's quality and/or effectiveness. Tell us about these changes and how would you use them? | 59 | 0.88 |  | Cargo et al - Edited as per feedback |
| You considered how many patients received the service elsewhere. How and when would you find out and how would it affect your consideration? | 60 | 0.75 |  | Cargo et al - Edited as per feedback |
| You considered how many ineligible patients received the service or should have received the service but did not. What steps would you take to avoid this issue? | 61 | 0.78 |  | Cargo et al - Edited as per feedback |
| You considered a patient satisfaction survey/interview. How would these findings inform service delivery? | 62 | 0.78 |  | Cargo et al - Edited as per feedback |
| You considered pharmacist satisfaction survey/interview about the service they delivered. How would these findings inform service delivery? | 63 | 0.75 |  | Cargo et al - Edited as per feedback |
| You considered what support the pharmacist (who delivers the service) receives from close colleagues. How would these findings inform service delivery? | 64 | 0.75 |  | Cargo et al - Edited as per feedback |
| You considered the amount of support pharmacist (who delivers the service) receives from allied health care providers. How would these findings inform service delivery? | 65 | 0.63 |  | Cargo et al - Edited as per feedback |
| Would you find it helpful if you had a manual on how to plan, implement and evaluate community pharmacy services - a guide with a checklist? | 66 | 0.78 |  | Cargo et al - Edited as per feedback |
