# Supplementary material for: Implementation priorities in Australian community pharmacy: A semi-structured survey of Australian pharmacists
Source: Explor Res Clin Soc Pharm. 2025 Nov 15;21:100683. doi: 10.1016/j.rcsop.2025.100683 (PMC12686930; doi:10.1016/j.rcsop.2025.100683)
Supplement: Supplementary file 2 — Adaptation of the twelve key dimensions of implementation by Cargo et al to pharmacy context [file mmc2.docx]

**Additional File 1.** Adaptation of the twelve key dimensions of implementation by Cargo et al to pharmacy context [1].

| Key dimension [1] | Adapted implementation dimension to the study context | Community pharmacy related operational definition | Adapted survey questions | |
| --- | --- | --- | --- | --- |
|  |  |  | **Quantitative survey question**  *Response style: 5-point Likert scale rating importance* | **Qualitative survey question**  *Response style: Free-text* |
| Dose delivered | Service availability | Amount of time the service was available for | The time the service takes for each person, e.g. ‘Service A’ will take 30 minutes per person | How would you estimate this number? |
| Dose received | Service uptake | Number of patients received the service | The number of people who responded to the invitation for the service | How would you estimate this number? |
| Reach | Eligible patients | Number of patients who are eligible for the service | The number of people that you could market the service to (reach) | How would you record this? |
| Recruitment | Patients approached | Number of patients approached per type of recruitment strategy | The number of people that would actually use the service | How would you record this? |
| Fidelity | Protocol application | Degree to which service delivery followed the protocol | The existing standard operating procedures (SOP), QCPP processes or internal work instructions for this service support its delivery | How would you (if applicable) develop the protocol? |
| Adaptation | Changes to service | Aspects of service delivery were intentionally changed by the pharmacist to enhance outcomes | Use of a quality improvement processes implemented by a pharmacist | Tell us about these changes and how would you apply them? |
| Cointervention | Other services/factors | Number/percentage of patients who received the service (or similar service which nulls this service) by other pharmacists/allied health | The number of people that have received the service elsewhere | How and when would you find out, and how would it affect your consideration? |
| Contamination | Unintentional service delivery | Number of patients receiving the service who should not have received the service | The number of people that may be accidentally provided (or not provided) the service | What steps would you take to avoid this issue? |
| Participant engagement | Patient experience | The degree of the patient’s satisfaction with the overall service | Patient satisfaction survey/interview of service delivery | How would these findings inform service delivery? |
| Implementer engagement | Pharmacist experience | The degree of the pharmacist’s satisfaction with the overall service | Pharmacist satisfaction survey/interview of service delivery | How would these findings inform service delivery? |
| Intervention quality | Resources quality | The degree of the pharmacist’s satisfaction with resource quality e.g. Training, guidelines, software support, materials | The level of support you (the pharmacist) would receive from other pharmacy staff to support service delivery regarding the service delivery | How would these findings inform service delivery? |
| Context | Internal service support | The degree to which partnerships with internal health care professionals supported the pharmacist in service delivery (eg. Other pharmacy staff) | The level of support you (the pharmacist) receive from external organisations and stakeholders (GPs, local optometrists, banner group) to support service delivery | How would these findings inform service delivery? |

1. Cargo, M., et al., *Cochrane Qualitative and Implementation Methods Group guidance series-paper 4: methods for assessing evidence on intervention implementation.* J Clin Epidemiol, 2018. **97**: p. 59-69.
