# Supplementary material for: Implementation priorities in Australian community pharmacy: A semi-structured survey of Australian pharmacists
Source: Explor Res Clin Soc Pharm. 2025 Nov 15;21:100683. doi: 10.1016/j.rcsop.2025.100683 (PMC12686930; doi:10.1016/j.rcsop.2025.100683)
Supplement: Supplementary file 3 — Mapping the implementation of professional services in Australian community pharmacy questionnaire [file mmc3.pdf]

Mapping the implementation of professional services in Australian community pharmacy questionnaire

STUDY NAME:

Mapping the implementation of professional services in Australian community pharmacy questionnaire

PARTICIPANT INFORMATION STATEMENT – SURVEY (PROVIDED ONLINE)

What is this study about?

You are invited to take part in a research study that maps the implementation of pharmacist delivered professional services in Australian community pharmacy.

You are invited to participate in this study because you are a registered community pharmacist practising in Australia, and your opinion matters. This Participant Information Statement tells you about the research study. Knowing what is involved will help you decide if you want to take part in the research. Please read this sheet carefully and ask questions about anything you do not understand or would like to know more about.

By giving your consent to take part in this study, you are telling us that you:

Understand what you have read.

Agree to take part in the research study as outlined below.

Agree to the use of your personal information as described.

You will be given a printed copy of this Participant Information Statement to keep upon request.

Who is running the study?

The following researchers are carrying out the study:

Veronika Seda, The University of Sydney, School of Pharmacy

Dr Carl Schneider, The University of Sydney, School of Pharmacy

Associate Professor Rebekah Moles, The University of Sydney, School of Pharmacy

Dr Stephen Carter, The University of Sydney, School of Pharmacy

Ms Veronika Seda is conducting this study as the basis for the degree of Doctor of Philosophy (Pharmacy) at The University of Sydney. This will take place under the supervision of Drs Schneider, Carter and A/Prof Moles.

Rebekah Moles is a Branch Committee member, and Veronika Seda is a Vice-President of their local branch at the Pharmaceutical Society of Australia (PSA).

This study is not funded.

What will the study involve for me?

This study called ‘Mapping the implementation of professional services in Australian community pharmacy questionnaire’ explores how Australian community pharmacists perceive the planning, implementation, and evaluation of professional services in community pharmacy.

This information gathered from the survey is part of a broader Doctor of Philosophy (PhD) project, which aims to develop a sustainable guide for delivery of professional services in community pharmacy in Australia.

You will not be asked any personal questions, and upon final submission of the completed form, there will be no way to identify your responses. Your responses will be voluntary and non-identifiable.

How much of my time will the study take?

The survey is designed to take no more than 20 minutes of your time and it is to be completed once only.

After the completion and submission of the form, no more of your time will be required.

Who can take part in the study?

Participation in this study is restricted to all currently registered community pharmacists in Australia.

Do I have to be in the study? Can I withdraw from the study once I've started?

Your participation in the study is voluntary, and your decision to participate in the study, or if required to withdraw during the study, will not have any impact on personal and professional relationships with either individual researchers or the team.

If you decide to participate in the study, submitting your completed questionnaire is an indication of your consent to participate in the study. You can withdraw your responses at any time before you have submitted the questionnaire. Once you have submitted it, your responses cannot be withdrawn because they are anonymous, and therefore, we will not be able to tell which one is yours.

Are there any risks or costs associated with being in the study?

Aside from giving up your time, we do not expect that there will be any risks or costs associated with taking part in this study.

Are there any benefits associated with being in the study?

We cannot guarantee that you will receive any direct benefits from being in the study. Your participation in this study may benefit future community pharmacists by providing feedback on professional services' implementation.

What will happen to information about me that is collected during the study?

The following types of information will be collected and used as part of this study:

Basic demographic data, such as gender, age, postcode of your practice, pharmacy type

You will be able to leave your contact details should you wish to be contacted for future study by the research team.

You will be able to leave your contact details should you wish to receive feedback about this study.

By providing your consent, you agree to assist us in the research study – a questionnaire called Mapping the implementation of professional services in the Australian community pharmacy questionnaire. The answers you will provide will only be used for the purposes outlined in this Participant Information Statement unless you consent otherwise.

Your personal details, such as name, email, and/or telephone number, will not be shared outside of the project and will remain securely protected by the University of Sydney. Further, the data you submit via the questionnaire will be non-identifiable, and any contact details provided will be stored separately from survey responses to maintain anonymity.

Results from this study may be published in scientific journals, conference presentations, briefs, and factsheets.

All electronic and hardcopy data collected as part of this study will be stored during and after the study in an electronic format in a password protected folder on the University of Sydney research drive. Only the investigators of this study will have access to this data. This data will be kept for a maximum of 5 years for the purposes of this research.

Can I tell other people about the study?

Yes, you are welcome to tell other pharmacists about the study.

What if I would like further information about the study?

If you would like to know more at any stage during the study, please feel free to contact Veronika Seda at vsed0158@uni.sydney.edu.au or Dr Carl Schneider at carl.schneider@sydney.edu.au.

Will I be told the results of the study?

You have a right to receive feedback about the overall results of this study. You can tell us that you wish to receive feedback by requesting it at the same time you return your online survey. There will be an opportunity to leave your email for this purpose.

What if I have a complaint or any concerns about the study?

Research involving humans in Australia is reviewed by an independent group of people called a Human Research Ethics Committee (HREC). The HREC of the University of Sydney has approved the ethical aspects of this study 2021/170. As part of this process, we have agreed to carry out the study according to the National Statement on Ethical Conduct in Human Research (2007). This statement has been developed to protect people who agree to take part in research studies.

If you are concerned about how this study is being conducted or wish to make a complaint to someone independent from the study, please contact the university using the details outlined below. Please quote the study title and protocol number.

The Human Ethics Manager, University of Sydney:

Telephone: +61 2 9036 9161

Email: ethics@sydney.edu.au

What type of registration do you currently hold with the Australian Health Practitioner Regulation Agency (AHPRA)?

- ◯ General registration
- ◯ Provisional registration
- ◯ Non-practising registration
- ◯ Limited registration
- ◯ Student registration

(For more information about the AHPRA registration types, please refer to this link: https://www.pharmacyboard.gov.au/registration.aspx)

In the last 14 days, have you spent more than SIX hours as a community pharmacist in Australia?

- ◯ Yes
- ◯ No

Which option best describes your role during this time?

- ◯ Pharmacist Owner
- ◯ Senior pharmacist/ Pharmacist manager
- ◯ Dispensing pharmacist
- ◯ Professional Services Pharmacist
- ◯ Dispensing and Professional Services pharmacist
- ◯ Compounding pharmacist
- ◯ Other

Please specify

____

Where is the pharmacy location where you have worked most in the last 14 days?

- ◯ Standalone pharmacy
- ◯ Strip of shops
- ◯ Shopping center
- ◯ Pharmacy located in general practice
- ◯ Other

Please specify

____

What is this pharmacy's postcode?

____

What type of pharmacy is this?

- ◯ Independent pharmacy
- ◯ Pharmacy is part of a banner group
- ◯ Other

Please specify

____

On average, how many pharmacists work in this pharmacy at this time? (Please consider a full-time equivalent)

One pharmacist — Two pharmacists — Four or more pharmacists

(Place a mark on the scale above)

How many intern pharmacists are currently working in this pharmacy? (Please consider a full-time equivalent)	__________________________
On average, how many prescriptions does the pharmacy dispense per day?	__________________________
How many hours were you involved in professional pharmacy services* in the last 14 days?	◯ Nil<br>◯ 1 to 5<br>◯ 6 to 10<br>◯ 10 or more<br>(In the context of this survey, professional pharmacy services are provided to a patient/carer inside of pharmacy, beyond traditional dispensing and medicine counselling, i.e. MedsCheck, immunisations, opioid substitution, etc.)
What was the reason you were not involved in professional pharmacy services in the last 14 days?	________________________________

The following questions relate to the community pharmacy, where you spent the most of your time in the last 14 days.

What professional pharmacist services were you involved in?

	Involved	Not involved
MedsCheck	◯	◯
Diabetes MedsCheck	◯	◯
Immunisations (except COVID-19 vaccinations)	◯	◯
COVID-19 vaccinations	◯	◯
Dose administration aids	◯	◯
Opioid substitution	◯	◯
Take-home naloxone medication service	◯	◯
Blood pressure measurement	◯	◯
Absence from work certificates	◯	◯
Weight management	◯	◯
Wound care	◯	◯
Other service	◯	◯

Please specify ________________________________

How many hours do you estimate you spent planning MedsCheck? ____________________

How many hours do you estimate you spent planning Diabetes Medscheck? ____________________

How many hours do you estimate you spent planning immunisations (excluding COVID-19 vaccinations)? ____________________

How many hours do you estimate you spent planning for COVID-19 vaccinations? ____________________

How many hours do you estimate you spent planning for dose administration aids' preparation? ____________________

How many hours do you estimate you spent planning for opioid substitution? ____________________

How many hours do you estimate you spent planning for the Take-home naloxone service? ____________________

How many hours do you estimate you spent planning for performing a blood pressure measurement? ____________________

How many hours do you estimate you spent planning for absence from work certificates?	______________________
How many hours do you estimate you spent planning for weight management services?	______________________
How many hours do you estimate you spent planning for wound care services?	______________________
How many hours do you estimate you spent planning for the other service?	______________________
To how many people did you personally deliver MedsCheck?	______________________
To how many people did you personally deliver Diabetes MedsCheck?	______________________
To how many people did you personally deliver immunisations (excluding COVID-19 vaccinations)?	______________________
For how many people did you personally prepare dose administration aids?	______________________
To how many people did you personally deliver opioid substitution?	______________________
To how many people did you personally deliver Take-home naloxone service?	______________________
For how many people did you personally perform a blood pressure measurement?	______________________
To many people did you personally counsel on the absence from work certificates?	______________________
To how many people did you personally provide weight management services?	______________________
To how many people did you personally provide wound care services?	______________________
To how many people did you personally deliver the other service you indicated?	______________________
To how many people did you personally deliver a COVID-19 vaccination?	______________________
How confident in your knowledge and skills did you feel when providing MedsChecks?	Not at all confident — Extremely confident<br>*(Place a mark on the scale above)*
How confident in your knowledge and skills did you feel when providing Diabetes MedsChecks?	Not at all confident — Extremely confident<br>*(Place a mark on the scale above)*

How confident in your knowledge and skills did you feel when providing immunisations?

Not at all confident — Extremely confident

(Place a mark on the scale above)

How confident in your knowledge and skills did you feel when providing dose administration aids?

Not at all confident — Extremely confident

(Place a mark on the scale above)

How confident in your knowledge and skills did you feel when providing opioid substitution?

Not at all confident — Extremely confident

(Place a mark on the scale above)

How confident in your knowledge and skills did you feel when providing Take-home naloxone medication service?

Not at all confident — Extremely confident

(Place a mark on the scale above)

How confident in your knowledge and skills did you feel when providing blood pressure management?

Not at all confident — Extremely confident

(Place a mark on the scale above)

How confident in your knowledge and skills did you feel when providing absence from work certificates?

Not at all confident — Extremely confident

(Place a mark on the scale above)

How confident in your knowledge and skills did you feel when providing weight management services?

Not at all confident — Extremely confident

(Place a mark on the scale above)

How confident in your knowledge and skills did you feel when providing wound care?

Not at all confident — Extremely confident

(Place a mark on the scale above)

How confident in your knowledge and skills did you feel when providing the other service?

Not at all confident — Extremely confident

(Place a mark on the scale above)

How confident in your knowledge and skills did you feel when providing COVID-19 vaccinations?

Not at all confident — Extremely confident

(Place a mark on the scale above)

The following questions relate to the community pharmacy, where you spent the most of your time in the last 14 days.

What communication processes does this pharmacy have for staff to provide service continuity? For example, how does the 'Sunday team' communicate with the 'Monday team'?

- ☐ Individual verbal communication
- ☐ Group verbal communication, e.g. meetings
- ☐ Handwritten notes, e.g. communication book
- ☐ Pharmacy software messages, e.g. dispensing software
- ☐ Private social media group communication, e.g. WhatsApp
- ☐ Google groups
- ☐ Other

Please specify

____

If it was up to you, which community pharmacy service, delivered by a pharmacist, you would most prefer to offer?

- ◯ MedsCheck
- ◯ Diabetes MedsCheck
- ◯ Immunisations (excluding COVID-19 vaccinations)
- ◯ COVID-19 vaccination
- ◯ Dose administration aids
- ◯ Opioid substitution
- ◯ Take-home naloxone medication service
- ◯ Blood pressure management
- ◯ Absence from work certificates
- ◯ Weight management
- ◯ Wound care services
- ◯ Other

Please specify

____

Why have you prioritised this service?

____

Have patients ever paid 'out of pocket' for professional service(s) in the pharmacy you worked most in the last 14 days?

For example, some services are offered as ‘free of charge’ in some pharmacies, but some pharmacies choose to charge patients ‘out of pocket’, e.g. blood pressure monitoring, wound care, staged supply, to name a few.

- ◯ Yes
- ◯ No

Please tell us how the service fee was calculated and how much the patients were charged.

____

What, in your opinion, is the reason some pharmacies provide services ‘free of charge’ when they could charge patients an ‘out of pocket’ fee for their provision?

____

Please tick one or more operating procedures or internal work instructions for the pharmacy, where you worked in the last 14 days.	☐ Quality Care Pharmacy Program (QCPP)<br>☐ Work instructions developed by this pharmacy staff<br>☐ Work instructions developed by the pharmacy banner group<br>☐ Other
Please specify	__________________________________
What training and other educational support, e.g. mentoring, has been available to you in relation to professional pharmacy services?	__________________________________
To your knowledge, before introducing any of the services in the pharmacy, was there any piloting or testing performed in this pharmacy (for example, testing the service with a mock patient to gain staff confidence, to test the process)?	◯ Yes, we tested the service before launching<br>◯ No, testing was not considered<br>◯ No, but testing was considered<br>◯ Unsure<br>◯ Other
Can you tell us how was this done?	__________________________________
Please specify	__________________________________

Please think about the community pharmacy, where you spent most of your time in the last 14 days. If you were to introduce a new professional service, how would you rate the importance of each of the following factors in facilitating the successful implementation? The word 'important' represents your own perception of how significant, useful, beneficial or necessary the individual aspect of implementation is.

Please rate the importance of the following criteria when considering implementing professional service.

	Not at all important	Slightly important	Important	Fairly Important	Very important	No opinion
The pharmacy has a plan, which outlines promotional activities for this new service	○	○	○	○	○	○
The pharmacy's investment into the new service is financially viable	○	○	○	○	○	○
Patients return to the pharmacy because of this new professional service	○	○	○	○	○	○
The new service you are about to start has been successfully trialed elsewhere	○	○	○	○	○	○
The amount of time the pharmacy staff has allocated to the service'	○	○	○	○	○	○
The pharmacists and assistants have the requisite knowledge and clinical skills related to this service before it is implemented	○	○	○	○	○	○
The local community sees this new service as needed	○	○	○	○	○	○
Before the new service is commenced, the pharmacy has the requisite equipment, such as necessary software, consultation room, operational procedures, materials, ready	○	○	○	○	○	○
Local health providers support the new service, e.g. if your pharmacy will be performing an eye screening, your neighbouring optometrist and GP support this service	○	○	○	○	○	○

The pharmacists practise to their full scope. NB: Expanded pharmacy services have been defined as working to their 'full' or 'enhanced' scope of practice, involving the performing of activities usually provided by other health professionals.	○	○	○	○	○	○
Your manager/s support you in implementing a new service	○	○	○	○	○	○
The service is supported by the IT technology in your pharmacy	○	○	○	○	○	○
You are supported by external organisations in implementing this new service, for example by a professional organisation launching a promotional campaign	○	○	○	○	○	○
This new service considers partnerships with the local community	○	○	○	○	○	○
The service is funded, e.g. someone pays for the service and is not provided by the pharmacy as 'free of charge'	○	○	○	○	○	○

Who decides on the services to be implemented in this pharmacy?

- ☐ Pharmacy owner
- ☐ Senior pharmacist/ Pharmacist manager
- ☐ Pharmacist (dispensing, professional services, compounding, etc)
- ☐ Other

Please specify

____

For the services provided in the pharmacy, are any of the pharmacy staff members assigned to be 'in charge' of the service?

- ○ Yes, formally, it is in their contract
- ○ Yes, informally, it is one pharmacist responsible for all pharmacy services' delivery
- ○ Yes, informally, that is the pharmacist on duty
- ○ No, the service is not assigned to anyone
- ○ Other

Please specify

____

What role does this person have in the pharmacy?

- ☐ Pharmacy owner
- ☐ Senior pharmacist/Pharmacist manager
- ☐ Pharmacist (dispensing, professional services, compounding, etc)
- ☐ Other

Please specify	__________________________________
Following the implementation of the service, when is the service delivered?	◯ The service is delivered when the opportunity arises ('ad hoc')<br>◯ The pharmacy books patients/clients in advance for the service<br>◯ Combination of 'ad hoc' and planned booking depending on staff availability<br>◯ Unsure<br>◯ Other
Please specify	____
The pharmacy is considering launching a new service. How is the service incorporated into existing work requirements?	☐ New staff are recruited to cover for when the pharmacist is providing the service<br>☐ Other services are discontinued in order to provide the new service<br>☐ No activities are undertaken, the new service is delivered in addition to other services provided<br>☐ Unsure<br>☐ Other
Please specify	____

The following questions refer to key factors of implementation. Please relate to the community pharmacy where you spent most of your time in the last 14 days.

Thinking about a future professional service you wish to provide, how important are the following implementation factors during your consideration?

	Not at all important	Slightly important	Important	Fairly important	Very important	No opinion
The time the service takes for each person, e.g. 'Service A' will take 30 minutes per person	○	○	○	○	○	○
The number of people who responded to the invitation for the service	○	○	○	○	○	○
The number of people that you could market the service to (reach)	○	○	○	○	○	○
The number of people that would actually use the service	○	○	○	○	○	○
The existing standard operating procedures (SOP), QCPP processes or internal work instructions for this service support its delivery	○	○	○	○	○	○
Use of a quality improvement processes implemented by a pharmacist	○	○	○	○	○	○
The number of people that have received the service elsewhere	○	○	○	○	○	○
The number of people that may be accidentally provided (or not provided) the service	○	○	○	○	○	○
Patient satisfaction survey/interview of service delivery	○	○	○	○	○	○
Pharmacist satisfaction survey/interview of service delivery	○	○	○	○	○	○
The level of support you (the pharmacist) would receive from other pharmacy staff to support service delivery regarding the service delivery	○	○	○	○	○	○

The level of support you (the pharmacist) receive from external organisations and stakeholders (GPs, local optometrists, banner group) to support service delivery	○	○	○	○	○	○

You would consider how much time is to be allocated to the service per patient. How would you estimate this number? __________________________________

You would consider how many patients responded to the invitation for the service. How would you estimate the number? __________________________________

You would consider how many patients, who visit this pharmacy, fit the criteria for the service. How would you estimate this number? __________________________________

You would consider how many patients were approached per type of marketing strategy (reach), e.g. pharmacist approached, flyers handed , etc. How would you record this? __________________________________

You would consider existing standard operating procedures (SOP), quality care pharmacy program checklist (QCPP) processes or internal work instructions for this service. How would you (if applicable) develop the protocol? __________________________________

You would consider the use of quality improvement processes implemented by a pharmacist in professional services. Tell us about these changes and how would you apply them? __________________________________

You would consider how many patients may have already received the service elsewhere. How and when would you find out, and how would it affect your consideration? __________________________________

You would consider how many people received the service but should not have and/or were eligible for the service but not included or identified. What steps would you take to avoid this issue? __________________________________

You would consider a patient satisfaction survey/interview. How would these findings inform service delivery? __________________________________

You would consider pharmacist satisfaction survey/interview about the service they delivered. How would these findings inform service delivery? __________________________________

You would consider the support the pharmacist receives from pharmacy staff for service delivery. How would these findings inform service delivery? __________________________________

You would consider the support pharmacist receives from external organisations and stakeholders (GPs, local optometrists, banner group) for service delivery. How would these findings inform service delivery?

____

How helpful would a manual or practical guide for everyday use in your practice on how to plan, implement and evaluate community pharmacy services be for you? This document would provide you with the skills to assess, plan, deliver and evaluate services' viability, quality and education.

- ◯ Not at all helpful
- ◯ Slightly helpful
- ◯ Moderately helpful
- ◯ Very helpful
- ◯ No opinion

Please tell us what content would you find valuable in such a step-by-step guide?

____

These last six questions focus on demographics.

What is your gender?

- ◯ Male
- ◯ Female
- ◯ Other
- ◯ Prefer not to say

What is your age?

____

Did you obtain your initial pharmacist qualifications in Australia?

- ◯ Yes
- ◯ No

Which of the following additional qualifications do you hold?

- ☐ AACP* accreditation
- ☐ Diabetes Educator
- ☐ Immunisation training
- ☐ Mental Health training
- ☐ Compounding course
- ☐ Other
- ☐ None
- ☐ Prefer not to say

(* Australian Association of Consultant Pharmacists)

Please specify

____

How many years have you been practising in Australian community pharmacy?

____

Would you like to leave your email to receive the information about the study?

Your email will be kept separate from the survey results, remaining anonymous.

If you would like to leave your email, please select 'Yes' from the options below.

- ◯ Yes
- ◯ No
